# Active Ageing Level of Older Persons: Regional Comparison in Thailand

**DOI:** 10.1155/2016/9093018

**Published:** 2016-06-07

**Authors:** Md. Nuruzzaman Haque

**Affiliations:** Department of Population Science and Human Resource Development, University of Rajshahi, Rajshahi 6205, Bangladesh

## Abstract

Active ageing level and its discrepancy in different regions (Bangkok, Central, North, Northeast, and South) of Thailand have been examined for prioritizing the policy agenda to be implemented. Attempt has been made to test preliminary active ageing models for Thai older persons and hence active ageing index (AAI, ranges from 0 to 1) has been estimated. Using nationally representative data and confirmatory factor analysis approach, this study justified active ageing models for female and male older persons in Thailand. Results revealed that active ageing level of Thai older persons is not high (mean AAIs for female and male older persons are 0.64 and 0.61, resp., and those are significantly different (*p* < 0.001)). Mean AAI in Central region is lower than North, Northeast, and South regions but there is no significant difference in the latter three regions of Thailand. Special emphasis should be given to Central region and policy should be undertaken for increasing active ageing level. Implementation of an Integrated Active Ageing Package (IAAP), containing policies for older persons to improve their health and economic security, to promote participation in social groups and longer working lives, and to arrange learning programs, would be helpful for increasing older persons' active ageing level in Thailand.

## 1. Introduction

Active ageing (AA) is the global goal of present ageing world for meeting the challenges of older persons and for improving their quality of life. Active ageing can be explained as a concept [1] and Walker (2002) has defined AA as profound strategy to maximize participation and wellbeing as people grow older [1]. World Health Organization (WHO) defined AA as the way of thinking and working on “the process of optimizing opportunities for health, participation and security in order to enhance quality of life as people age” [2]. The concept of AA can be encapsulated as “engaged in life” and it has been recognized as a latent construct which has no specific clear dependent variable to measure it and it may be determined by various latent (unobserved) factors. The AA construct is influenced by several groups of determinants or determinant factors including the cross-cutting determinants (gender and culture) [2]. These determinant factors are unobserved or latent and each of them can be presented by some indicators [2, 3].

The growth of population ageing in Thailand is faster compared to other Asian countries [4]. The pace of recent population ageing in Thailand is faster than other Asian countries and even far more faster than developed countries in the West [4, 5], and proportion of elderly (aged 60 and over) is projected to reach more than 30% within the next three decades in Thailand [6]. Thailand has become an ageing society and is going to increase its older population. A rapid proceeding pace of population ageing, along with demographic changes in Thailand, is posing critical challenges for Thai government to maintain economic growth, social stability, and living standard of people in the nation.

All determinant factors of AA with their indicators (hence active ageing level, AAL) need to be better understood for developing policies and programs focused on active ageing in an ageing society. Moreover, comparison of AAL regarding cross-cutting determinants of AA, gender and culture, should be unveiled for better policy implications for the older persons and for the nation as a whole. Using exploratory factor analysis, one study by Haque et al. found active ageing determinant factors models with six determinant factors for female and male older persons in Thailand [3]. But the study did not validate the models (i.e., how does the model fit with the sample data?). Along with the lack of model validation, the study by Haque et al. (or any other study so far in knowledge) did not compare AAL for culture of different regions in Thailand. Culture, defined as people's behavior, attitude, or way of life, varies depending on region of residence (e.g., urban or rural, different region of a country) of individuals. Therefore, people's behavior, attitude, or way of life depends on the place or region of society where the society is situated. Though Thailand is perceived as Southeast Asia's most ethnically homogeneous nation state and strong unity of Thai culture, Thailand government's use of regional terms (Central, North, Northeast, and South) indicates some regional cultural diversity in Thailand [7]. Despite the important strength and unity of Thai culture, each region in Thailand has its own unique cultural features [8]. So, doing research or formulating policies on active ageing in Thailand, the variations of active ageing depending on region of residence (Central, North, Northeast, and South) should be considered or should be borne in mind. Hence active ageing level should be compared in different regions of Thailand.

The objective of this study was to gain empirical knowledge on active ageing determinant factors model for Thai older persons including their active ageing level. The main specific objectives of this study are (i) to test the validity of active ageing determinant factors model for Thai older persons and (ii) to test the similarity of active ageing level for various regions (Bangkok, Central (excluding Bangkok), North, Northeast, and South) in Thailand.

## 2. Materials and Methods

### 2.1. Data

The data for this study come from a nationally representative sample from the 2011 Survey of Older Persons in Thailand (SOPT). The SOPT was conducted by Thailand's National Statistical Office (NSO). The sample of SOPT consists of 62,840 persons aged 50 years and over, 54% of whom were 60 years and older. The data have been collected in SOPT by using stratified two-stage sampling method. The primary sampling units were villages for nonmunicipal areas and blocks for municipal areas. The secondary sampling units were private dwellings selected by random sampling from the list of all enumerated households in each village or block of the first sampling units. The 2011 SOPT is a nationally representative survey which covers data for population subgroups or geographic subareas (e.g., regions and urban-rural areas). Further details of the methodology, including sampling strategy, for SOPT are available at NSO's “report on the 2011 Survey of Older Persons in Thailand” [9]. This study included individuals (same as in Haque et al. study [3]) aged 60 years and over (*n* = 23,801; female = 14,369 and male = 9,432).

### 2.2. Variables

For validating the active ageing determinant factors structure in Thailand (suggested by Haque et al.) [3], the variables used for this study in confirmatory factor analysis (CFA) are provided in Table 1.

### 2.3. Hypothesized Active Ageing Determinant Factors Model

**Figure 1:**
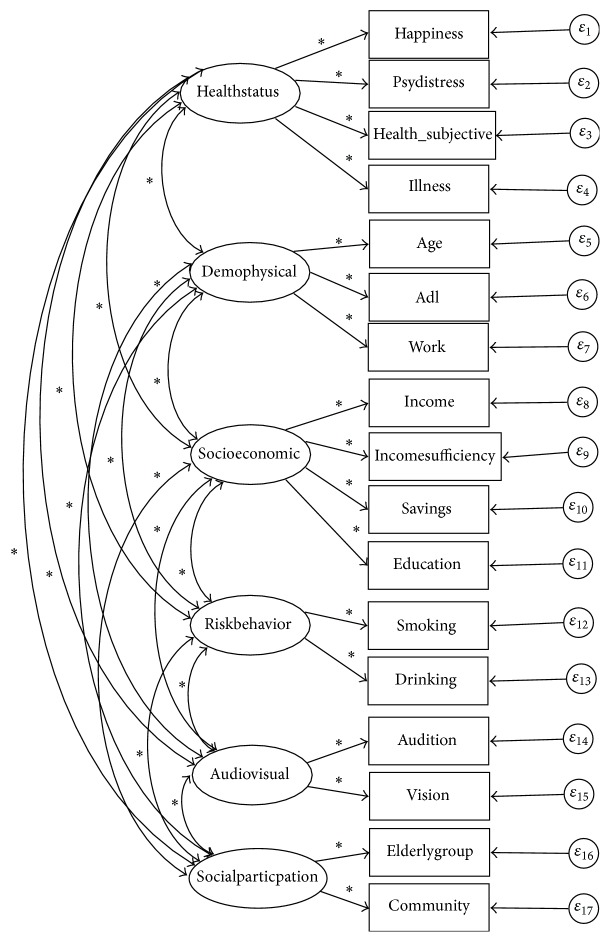
Hypothesized active ageing determinant factors model for female older persons in Thailand. Source: figure is constructed from Haque et al.'s study [3].

**Figure 2:**
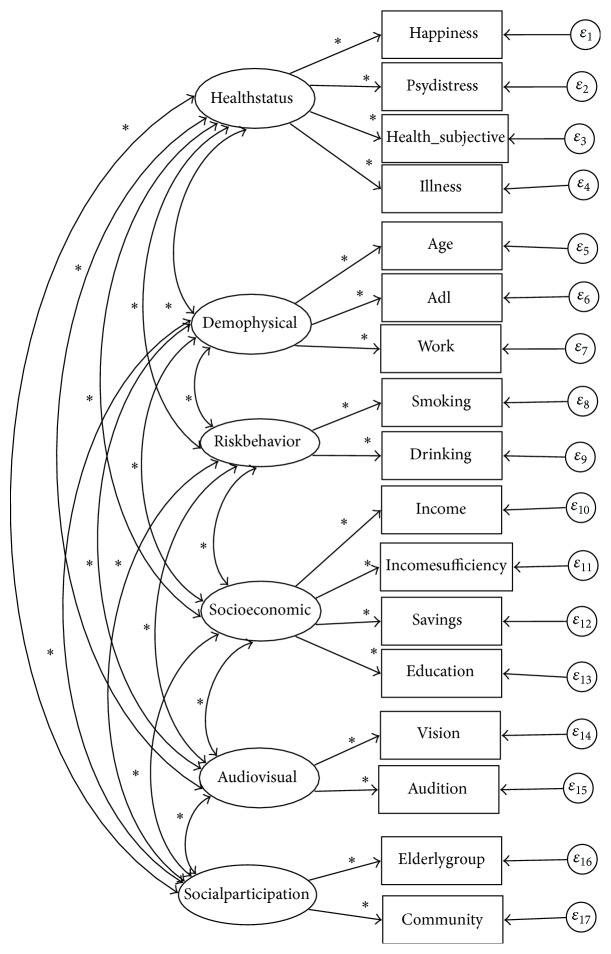
Hypothesized active ageing determinant factors model for male older persons in Thailand. Source: figure is constructed from Haque et al.'s study [3].

The study by Haque et al., a pioneer study for finding a model for active ageing determinants, suggested two separate determinant factor structures for each gender [3]. Using the indicator variables, listed in Table 1, the suggested active ageing determinant factors models of female and male older persons [3] are provided in Figures 1 and 2, respectively.

### 2.4. Description of Hypothesized Models Presented in Diagrams

In the hypothesized models in Figures 1 and 2, the factors are represented in ovals and indicator variables are represented in rectangles. The error terms of indicator variables are indicated by *ε*. The asterisks indicate parameters (regression coefficient (standardized), covariances of independent variables in the model) to be estimated. Meanwhile, error terms in CFA are considered as independent variables and their variances should be estimated [10]. Unidirectional arrows in the hypothesized models indicate directional linear influences (i.e., linear effect of one variable on another), and both-way directional arrows indicate correlation between connected variables but no directional influence of one on others is hypothesized.

### 2.5. Model Evaluation

Parameters of each of the hypothesized models can be estimated in CFA framework which was done using STATA 12 (model fit statistics, in CFA, are available after estimation of parameters). Maximum likelihood (ML) estimation method is currently one of the most used methods for parameter estimation [10]. Details of parameter estimation procedure in CFA are available elsewhere [10–12]. As this paper has no aim to estimate the parameters of those models (but has aim to evaluate those models), the parameter estimation results are not shown. Any estimated model should be tested for how well the model fits to the sample data. There are many fit indices for evaluating models in CFA framework. This study used Chi-square (*χ* ^2^), Root Mean Squared Error of Approximation (RMSEA), Comparative Fit Index (CFI), and Standardized Root Mean Squared Residual (SRMR). The *χ* ^2^ is a traditional measure of overall model fit (it assesses the magnitude of differences between sample and estimated covariance matrices) [13]. That is, the null hypothesis for model evaluation is as H_0_: there is no difference between sample covariance and estimated covariance; that is, the model fits well to the data. The threshold values of the above fit statistics are provided in Table 2.

### 2.6. Estimation of Active Ageing Level

Active ageing level can be exhibited by computing the active ageing index (AAI) of older persons [3]. The AAI ranges from 0 to 1 and a higher value of AAI indicates higher active ageing level. The study by Haque et al. (2016) has suggested that AAI should be calculated separately for female and male older persons in Thailand. Using nationally representative sample and exploratory factor analysis (EFA), Haque et al. calculated the AA level for female and male older persons in Thailand [3]. The weakness of results from a model constructed from EFA is that there is no scope to test the model fit. Haque and colleagues study used only EFA and the model (from which AAI has been obtained) was not validated. For overcoming the weakness in EFA, this study used confirmatory factor analysis (CFA). The CFA framework facilitates testing how well the model fits to the sample data. The study by Haque and his colleagues used EFA in Thai national data for establishing the preliminary active ageing model of older persons and the study has identified six determinant factors for each gender [3]. Using the same factors (with their corresponding indicator variables) as in Haque et al.'s study, this study runs the model in CFA framework (in STATA 12) which provides the predicted factor scores of each factor. From the predicted factor score, the factor index for each factor has been estimated using the following formula:

Index of F_*i*_:

(1) $$\begin{array}{c} f_{i}=\frac{\left[\text{Score of }{\text{F}}_{\text{i}}-\min\left(\text{Score of }F_{i}\right)\right]}{\left[\max\left(\text{Score of }F_{i}\right)-\min\left(\text{Score of }{\text{F}}_{\text{i}}\right)\right]}, \end{array}$$

where *F*_*i*_ is the *i*th factor.

Score of *F*_*i*_ for every individual has been produced during CFA running in STATA. The theoretical maximum value of any factor index is 1. Then an AAI was calculated by using factor specific indices. Calculation of the AAI, from CFA, used the following formula:

(2) $$\begin{array}{c} AAI=\frac{\sum_{i=1}^{n=6}f_{i}}{6}, \end{array}$$

where *f*_*i*_ is the index of factor *i* (*n* = 6, because there were 6 factors).

It should be noted here that the AAI has been calculated for every individual. Then an overall average AAI has been calculated using all values of AAI for all individuals. For simplicity, the AAI is a composite index which combines a number of factor indices.

### 2.7. Active Ageing Level Comparison Depending on Region of Residence

Because of social change over time and of cultural diversities in different places, possible changes may occur in individual AAL over time, as has occurred in other aspects of individual or population in Thailand; for example, between 1994 and 2007 Thailand experienced rapid social changes (population ageing, socioeconomic development, health policies, etc.) and these changes affect health across groups (rural-urban) and overall [15]. As has been stated in Introduction, there exists cultural variability in Thailand depending on region of residence (Bangkok, Central, North, Northeast, and South). It also may be hypothesized that under the circumstances of cultural diversities in different cultural settings, there would be variability of AA level in various cultural contexts, that is, in various regions within a country.

AA level quartiles would be the one method of comparing changes in the AAL at a community over time or for comparing it in different places or for groups. AA level quartiles can be calculated by ranking individuals from lowest AAI to the highest value and then dividing list into four groups or quartiles. Each quartile for individuals, in whole Thailand, for instance, contains 25% of individuals (along with specific mean level of AAI). Then we may compare those distributions of quartiles (percentage distribution) for individuals overall (national) with different places (e.g., Bangkok, Central, North, Northeast, and South). This quartile method of comparison will provide only the overview of difference(s) but is unable to test the significance of the differences. Similarity with significance of AA levels in different regions could be tested by using one-way analysis of variance (ANOVA). One-way ANOVA is appropriate for one categorical variable with *k* levels (e.g., region of residence with 5 levels in this study) or *k* groups and there should be a population of interest for which there is a quantitative variable for each of the *k* levels of the categorical variable (e.g., AAI in this study). The AAIs for each group have mean parameters (population means) that we may label as *μ*_1_ through *μ*_*k*_. The number of individuals (older persons) in group *i*  (1 ≤ *i* ≤ *k*) is defined as *n*_*i*_ and *n* = ∑_*i*=1_^*k*^ *n*_*i*_ is the total sample size. The null hypothesis to be tested in one-way ANOVA states that all the population means are equal; that is, H_0_: *μ*_1_ = *μ*_2_ = *μ*_3_ = *μ*_4_ = *μ*_5_ (for 5 regions of this study). The ANOVA technique can identify significant differences (if there were any) but is unable to specify where the significant differences exist. So, post hoc comparisons using a built-in test in STATA (e.g., Bonferroni test) were conducted.

## 3. Results and Discussion

### 3.1. Results

#### 3.1.1. Model Fit Statistics

It has been stated, in Materials and Methods, that the model fit statistics can be obtained after the model estimation in CFA. The hypothesized models have been run in CFA in STATA 12 and the obtained model fit indices are summarized in Table 3.

The Chi-square (*χ* ^2^) statistics for two models are very high and indicated that none of the models fits well to the sample data. But the *χ* ^2^ statistic is very sensitive to sample size; usually for large sample (which is the case for two models) it shows significant result which rejects the null hypothesis (H_0_: there is no difference between sample covariance and estimated covariance; i.e., the model fits well to the data). For correcting the sensitivity of *χ* ^2^ statistic to large sample (i.e., tendency to reject the null hypothesis), there are other test statistics which are most widely used (e.g., Root Mean Squared Error of Approximation (RMSEA), Comparative Fit Index (CFI), and Standardized Root Mean Squared Residual (SRMR)). However, comparing the estimated RMSEA, CFI, and SRMR fit indices presented in Table 3 with the threshold values of those indices presented in Table 2 suggests that both the estimated models seemed to fit well to the data. That is, active ageing determinant factors model for each gender can be adequately described by those six respective (to each model) correlated factors.

#### 3.1.2. Active Ageing Level for Female and Male Older Persons

As, in the Haque et al. study, relationships of indicator variables with determinant factors of active ageing (factor structure of active ageing, i.e., the active ageing determinant factors model) are different for female and male older persons [3], separate active ageing index has been estimated for female and male older persons. The calculated mean AAIs for female and male older persons are 0.64 and 0.61, respectively. Comparing these mean AAIs with UNDP's Human Development Index (HDI), female and male older persons in Thailand had moderate level mean AAI [16].

Female and male older persons have been divided into four groups according to their mean AAI (i.e., they are divided according to quartiles of mean AAI); mean AAI for each group has been presented in Table 4.

An independent-samples *t*-test was run to determine if there were differences in mean AAI of female and male older persons. This study found that active ageing level of female is bit higher than active ageing level of male older persons. The calculated statistic, *t*(23799) = 21.554 with *p* < 0.001, indicates a statistical significant difference of two populations' mean level of active ageing (may reject the null hypothesis that there is no difference between the mean of AAI of female and male older population). It implies that mean active ageing levels of female older population and male older population, in Thailand, are significantly different.

#### 3.1.3. Active Ageing Level Comparison Depending on Region of Residence

**Figure 3:**
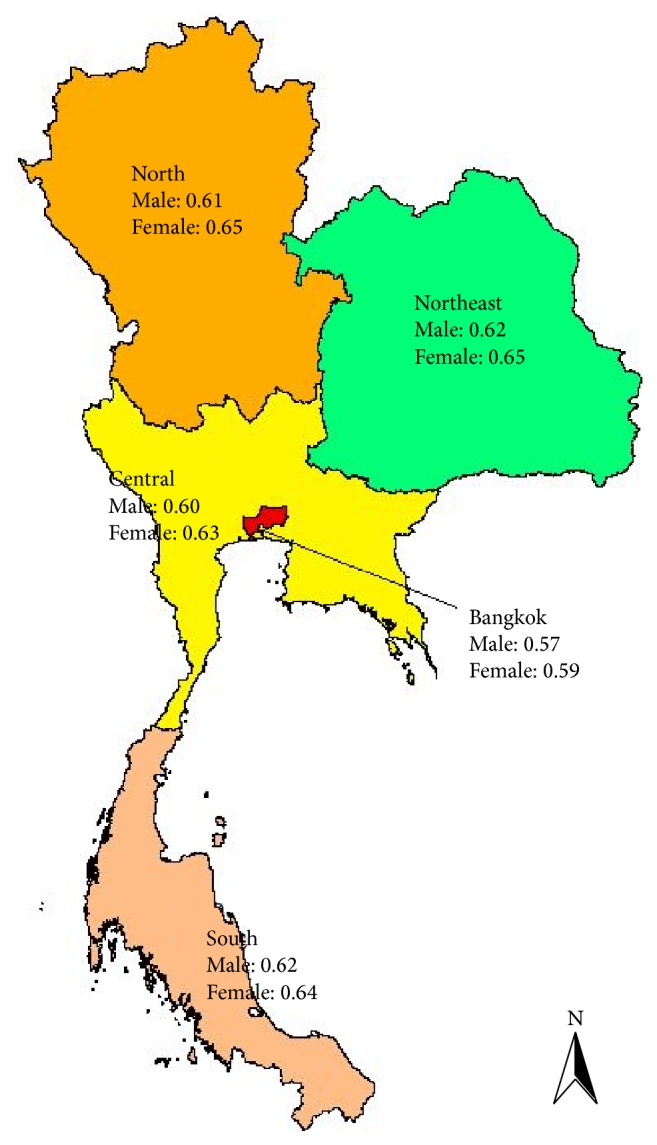
Map of Thailand with region-wise mean active ageing index value of female and male older persons.

The calculated average active ageing levels of female and male older persons for every five regions (Bangkok, Central, North, Northeast, and South) have been portrayed in Figure 3. Region-wise mean active ageing level of older persons represented in Figure 3 shows the glimpse of situation only. But distribution of older persons according to AAI quartiles and region of residence enables showing the apparent disparities of AAL in different regions. Distribution of older persons according to AAI quartiles and region of residence is provided in Tables 5 and 7.

The results in Table 5 exhibited that highest proportion of female older persons in Bangkok was the lowest group of mean AAI whereas in North and Northeast region highest proportion was in medium highest group of mean AAI. The highest percentage of female older persons in South region was in highest group of mean AAI. Female older persons from the North and Northeast region had equal mean AAI and higher mean AAI than other regions. Comparing with UNDP's HDI, overall mean AAIs of female elderly in all five regions were in moderate level [16].

A one-way ANOVA between female elderly persons was conducted to compare the mean AAI for regions (Bangkok, Central, North, Northeast, and South). There was a significant difference of mean AAI at the *p* < 0.001 level for the five regions [*F*(4,14364) = 56.79; *p* = 0.00]. The results of ANOVA found significant differences but could not specify where the significant differences exist. So, post hoc comparisons using Bonferroni test were conducted and the results are presented in Table 6.

Post hoc comparisons by Bonferroni test indicated that the mean AAI of female older persons is significantly lower in Bangkok than all other regions. Mean AAI of female older persons is significantly higher in Central region than Bangkok but the first two regions are lower than the other three regions (North, Northeast, and South). There is no significant difference in female elderly's mean AAI in North, Northeast, and South of Thailand.

The results in Table 7 revealed that highest proportion of male older persons in Bangkok and in Central region were in the lowest group of mean AAI whereas in North region highest proportion were in medium highest group of mean AAI. The highest percentage of male older persons in Northeast and in South region was in highest group of mean AAI. Overall, it can be mentioned from the sample results that male older persons from Northeast and South region had equal mean AAI and higher mean AAI than that of other regions. Comparing with UNDP's HDI, overall mean AAI male older persons in all five regions were in moderate level [16].

 A one-way ANOVA between male elderly persons was conducted to compare the mean AAI for regions (Bangkok, Central, North, Northeast, and South). There was a significant difference of mean AAI at the *p* < 0.001 level for the five regions [*F*(4,9427) = 28.58; *p* = 0.00]. The results of ANOVA found significant differences but could not specify where the significant differences exist. So, post hoc comparisons using Bonferroni test were conducted and the results are presented in Table 8.

Post hoc comparisons by Bonferroni test indicated that the mean AAI of male older persons is significantly lower in Bangkok than all other regions. Mean AAI of male older persons is significantly higher in Central region than Bangkok but the first two regions (Bangkok and Central) are lower than the other three regions (North, Northeast, and South). Male older persons' mean AAI is significantly similar in North, Northeast, and South region.

### 3.2. Discussion

As it has been stated and also proved that active ageing determinant factors model varies for female and male older persons, their active ageing level also varies; for instance, results revealed that the overall mean AAI for female and male older persons was revealed as 0.64 and 0.61, respectively. These estimates proved the hypothesis regarding the one cross-cutting determinant, gender, (i.e., active ageing determinant factors model and active ageing level vary depending on gender in Thailand) as true. The numerical values of AAI for both female and male older persons must aim for further improvement. Because active ageing index shows that even top performing female older persons still fall short by almost 36% to the highest desired status possible (i.e., the goalpost of AAI is equal to 1).

If we look at region-wise active ageing level, then it is evident that active ageing level of older persons in Central region is comparatively lower than other regions. Results for comparing active ageing level in different regions revealed that there were disparities in mean AAI for female older population. Female older persons from Bangkok region had the lowest level of mean AAI among the five regions. Even though mean AAI of female older persons in the three regions (North, Northeast, and South) seemed significantly the same, female older persons in Central region had significantly lower mean AAI than the North, Northeast, and South region. On the other hand, as stated earlier that overall estimated mean AAI of male older persons is lower than female older persons, it also revealed from the results that the above is also true for every five regions. Also, the test results for regional differences for mean AAI of male older persons follow the same pattern as for female older persons.

As active ageing level in Thailand is not high (evidenced as far behind the goal), so, for promoting active ageing, focus should be given to the identified determinant factors of active ageing. As determinant factors are latent, hence focus should be given to their corresponding indicator variables (i.e., measured variables) aiming to promote active ageing. To make results more understandable, relation of directly measured variables with active ageing level should be interpreted which may be helpful for other researchers and policy makers. Using measured variables for each model, correlations between AAI and measured variables could be estimated. Correlation coefficient between measured variables and AAI for older persons has been calculated in the study by Haque et al. (2016). As data are the same for this study and Haque et al.'s study, so recommendations depending on the results regarding correlation coefficients (as in Haque et al.'s study) [3] could be appropriate for this study also. Haque et al.'s study urged that good health condition, opportunities of longer working lives, continuous income, participation in social group(s), and lifelong learning influence active ageing among the Thai older persons [3]. So, an Integrated Active Ageing Package (IAAP) would be helpful for increasing older persons' active ageing level. The IAAP should include policies for older persons to improve their health and economic security, to promote participation in social groups and longer working lives, and to arrange learning programs.

## 4. Conclusions

As active ageing level of older persons in Thailand is not high, so, some policy recommendations should be considered to increase the active ageing level of future older persons. The Thai government's national policy on the elderly (NPE) should include a new strategy on increasing active ageing level of elderly. Special emphasis should be given to Central region, as older persons in Central region are lower in respect to active ageing level compared to other regions. Policy should be focused for older persons on improving health and economic security, on promoting participation in social groups and longer working lives, and on arranging education (e.g., lifelong learning) programs for increasing their active ageing level. All the above policies should be integrated into one package as Integrated Active Ageing Package (IAAP). Along with the policy for extension of compulsory retirement age, incentives for elderly workers, and incentives for employers (who employed older persons), the IAAP should include some programs. The IAAP programs may include health programs—curative care for illness and continuous mental health care, easy access for assistive devices (e.g., walker/mover, eye glasses), and preventive and promotive health measures such as exercise and annual health checkup; community and elderly group participation programs; tobacco cessation counseling program; special knowledge and information for quality of life development program for elderly; and vocational training (including occupational retraining) program.

The proposed strategy, for NPE, on increasing active ageing level of older persons can be fulfilled by implementing the IAAP. The Ministry of Social Development and Human Security (MoSDHS), Thailand, may lead the implementation of IAAP. The MoSDHS may collaborate with the Ministry of Health and the Department of Non-Formal Education for full implementation of those programs. Implementation of IAAP would be helpful for increasing active ageing level of older persons which, in turn, is helpful for prolonging good health and independent living of older persons, that is, for increasing the quality of older persons' lives.

## Figures and Tables

**Table 1:** Selected indicator variables for validating active ageing determinant factors model in Thailand.

Variables	Coding
Age	1: 60–69 years; 2: 70–79 years; 3: 80+ years
Happiness level^a^	1: 1–6; 2: 7-8; 3: 9; 4: 10
Psychological distress status	1: very high; 2: high; 3: moderate; 4: poor or nearly never
Smoking	0: yes; 1: no
Drinking alcohol	0: yes; 1: no
Basic ADL (activities of daily living) index	0: severe; 1: moderately severe; 2: moderate; 3: independent
Subjective health	0: poor; 1: moderate; 2: good
Illness	0: two and/more chronic illnesses; 1: one chronic illness; 2: none
Visibility	0: no or not clear; 1: clear
Hearing	0: no or not clear; 1: clear
Education	0: no or less than primary; 1: primary; 2: secondary and/or higher
Community participation	0: no; 1: yes
Participation in elderly group	0: no; 1: yes
Work	0: no; 1: yes
Income^a^	1: no or <20000 Baht; 2: 20000–40000 Baht; 3: 40000–60000 Baht; 4: 60000+ Baht
Sufficiency of income	0: not sufficient; 1: sufficient
Savings	0: no; 1: yes

^a^Quartiles.

**Table 2:** Some selected model fit indices and their cut-off points (limits).

Fit index	Acceptable threshold levels
Chi-square (*χ* ^2^) (df, *p* value)	Low *χ* ^2^ relative to degrees of freedom (df) with an insignificant *p* value (*p* > 0.05)
Root Mean Squared Error of Approximation (RMSEA)	Values less than 0.07
Comparative Fit Index (CFI)	Closer to 1 is better but ≥0.90 indicates good fit
Standardized Root Mean Squared Residual (SRMR)	<0.05

Source: [14].

**Table 3:** Goodness of model fit indices for confirmatory factor analysis for models of active ageing for older persons in Thailand.

Fit index	Female	Male
Chi-square (*χ* ^2^) (df, *p* value)	3548.33 (104, 0.00)	3131.96 (104, 0.00)
Root Mean Squared Error of Approximation (RMSEA)	0.048	0.056
Comparative Fit Index (CFI)	0.867	0.839
Standardized Root Mean Squared Residual (SRMR)	0.035	0.043

**Table 4:** Mean level of active ageing of older persons in Thailand.

	Mean of AAI	
	Female	Male
Lowest group	0.49	0.46
Medium lowest	0.61	0.58
Medium highest	0.69	0.66
Highest group	0.77	0.74
Overall mean AAI	0.64	0.61

*t* = 21.554, *p* < 0.001.

**Table 5:** Distribution of female older persons according to quartiles of mean active ageing level and region.

Region	Bangkok	Central^*∗*^	North	Northeast	South	Total
*Total cases*	* 652*	* 4,706*	*3,560*	*3,496*	*1,955*	*14,369*
Lowest group	43.56	28.58	21.15	19.94	26.29	25.00
Medium lowest	30.98	25.18	24.02	25.69	23.12	25.00
Medium highest	09.05	22.16	29.80	28.86	21.48	25.00
Highest group	16.41	24.08	25.03	25.51	29.10	25.00
Overall mean AAI	0.59	0.63	0.65	0.65	0.64	0.64

^*∗*^Excluding Bangkok, *F*-statistic = 56.59, *p* < 0.001.

**Table 6:** Comparison of mean AAI of female older persons by region (Bonferroni test).

Row mean-col. mean	Bangkok	Central	North	Northeast
Central	0.04 (0.00)			
North	0.06 (0.00)	0.02 (0.00)		
Northeast	0.06 (0.00)	0.02 (0.00)	0.00 (1.00)	
South	0.05 (0.00)	0.01 (0.001)	−0.01 (0.291)	−0.01 (0.044)

*Note*. Numbers within bracket indicate the level of significance (the test is considered significant at <0.01).

**Table 7:** Distribution of male older persons according to quartiles of mean active ageing level and region.

Region	Bangkok	Central^*∗*^	North	Northeast	South	Total
*Total cases*	*394*	*2,914*	*2,558*	*2,206*	*1,360*	*9,432*
Lowest group	40.10	29.72	22.91	19.63	23.16	25.00
Medium lowest	29.95	24.09	23.18	28.42	23.38	25.00
Medium highest	14.21	22.82	29.52	25.43	23.60	25.00
Highest group	15.74	23.37	24.39	26.52	29.85	25.00
Overall AAI	0.57	0.60	0.61	0.62	0.62	0.61

*Note*. *F*-statistic = 28.58; *p* < 0.001; ^*∗*^excluding Bangkok.

**Table 8:** Comparison of mean AAI of male elderly by region (Bonferroni).

Row mean-col. mean	Bangkok	Central	North	Northeast
Central	0.03 (0.00)			
North	0.04 (0.00)	0.01 (0.00)		
Northeast	0.05 (0.00)	0.02 (0.00)	0.01 (1.00)	
South	0.05 (0.00)	0.02 (0.00)	0.01 (1.00)	0.00 (1.00)

*Note*. Numbers within bracket indicate the level of significance (the test is considered significant at <0.01).
